# Soft material for soft actuators

**DOI:** 10.1038/s41467-017-00685-3

**Published:** 2017-09-19

**Authors:** Aslan Miriyev, Kenneth Stack, Hod Lipson

**Affiliations:** Department of Mechanical Engineering, Columbia University in the City of New York, 500W 120th St., Mudd 220, New York, NY 10027 USA

## Abstract

Inspired by natural muscle, a key challenge in soft robotics is to develop self-contained electrically driven soft actuators with high strain density. Various characteristics of existing technologies, such as the high voltages required to trigger electroactive polymers ( > 1KV), low strain ( < 10%) of shape memory alloys and the need for external compressors and pressure-regulating components for hydraulic or pneumatic fluidicelastomer actuators, limit their practicality for untethered applications. Here we show a single self-contained soft robust composite material that combines the elastic properties of a polymeric matrix and the extreme volume change accompanying liquid–vapor transition. The material combines a high strain (up to 900%) and correspondingly high stress (up to 1.3 MPa) with low density (0.84 g cm^−3^). Along with its extremely low cost (about 3 cent per gram), simplicity of fabrication and environment-friendliness, these properties could enable new kinds of electrically driven entirely soft robots.

## Abstract (web summary)

The development of self-contained electrically driven soft actuators with high strain density is difficult. Here the authors show a single self-contained soft robust composite material that combines the elastic properties of a polymeric matrix and the extreme volume change accompanying liquid vapour transition.

## Introduction

Inspired by biology, researchers aim to develop soft-bodied programmable motion in order to combine natural compliance with controllable actuation. One of the long standing challenges has been the lack of easily processed robust soft actuators with high strain density^1–5^. Such actuators would be easy to produce and to mold, cut, and 3D print into a desired shape, yet would produce large macroscopic actuation at relatively low voltage and current. Today, soft actuation techniques are based on either electroactive polymers^6–12^, shape memory alloys and shape memory polymers^13–15^, or compressed air and pressurized fluids actuators^16–24^. However, the high voltages required to trigger electroactive polymers ( > 1KV) and low strain ( < 10%) of shape memory alloys, as well as the need for external compressors and pressure-regulating components for hydraulic^16, 18, 21, 24^ or pneumatic ^16, 17, 19, 20, 22, 23^ fluidic elastomer actuators, limit their miniaturization^2, 4, 16^ and practicality for untethered applications. Recent demonstrations of actuation based on combustion^25^ are ideal for impact delivery, but are less suitable for controllable kinematics.

Phase change materials offer an attractive alternative to conventional electromechanical actuators. Such materials rely on the mechanical force produced by the rapid expansion that occurs at the phase transition temperature. One of the classic examples of phase change materials is paraffin, which thermomechanical properties were first utilized in early 1930s^26^ for self-regulating vents in greenhouses.While paraffin-based actuators can deliver large forces, their strain remains in the order of 10% volumetric change^26–28^, a strain that is on par with shape memory alloys and too small for most robotics applications.

A significantly higher expansion strain may be achieved by utilizing reversible liquid–vapor phase transition, but such material systems have been traditionally difficult to contain and control. A number of recent devices use entrapped liquid inside balloons or between thin films, to form expanding cavities^29–32^. Electrically triggered deformation of soft elastomer membranes, utilizing liquid–gas transition of liquid, was reported to show large area expansion^33^. However, such devices are challenging to manufacture and to form into arbitrary shapes because of their intricate internal design. For example, it is difficult to directly cast or 3D-print any of these actuators.

Here we propose a single easily prepared soft robust material that combines the elastic properties of a polymeric matrix and the extreme volume change of a fluid upon liquid–vapor transition. We show and characterize the soft composite material comprised of a silicone elastomer matrix with ethanol distributed throughout it in micro-bubbles, exhibiting strains up to 900%, and demonstrate its use as an actuator in a range of robotic applications.

## Results

### Materials system and its principles of action

Choosing a polymer matrix and a fluid for the composite meta-material system was guided by the desired mechanical properties of a polymer, boiling point and practical handling restrictions of a fluid, and chemical compatibility of the two. We aimed to synthesize a cheap, simple, user- and environment-friendly material comprised of food-safe and bio-compatible materials. We chose PDMS-based silicone elastomer, a non-hazardous elastomer widely used for soft robotic applications, as a matrix material, and ethanol, a widely used alcohol with boiling temperature 78.4 °C and matrix-compatibility, as the active fluid (Supplementary Fig. 1 and Supplementary Discussion for a discussion of the material components choice).

Ethanol, included inside tiny micro-bubbles embedded in the elastic silicone rubber matrix, boils upon reaching the liquid–gas transition temperature, accompanied by tremendous increase in volume, leading to significant expansion of the whole soft composite material. This composite material may be quickly and easily prepared by mixing ethanol with silicone elastomer (Supplementary Movie 1, Supplementary Software 1). The mixed material is both castable and 3D-printable (Supplementary Movies 2, 4), and after preparation will solidify in room-temperature curing. We successfully mixed various amounts of ethanol (0–33 vol%) in the two-part platinum-catalyzed silicone elastomer (Supplementary Fig. 2). In total 20 vol% ethanol was chosen as optimal composition.

**Fig. 1:**
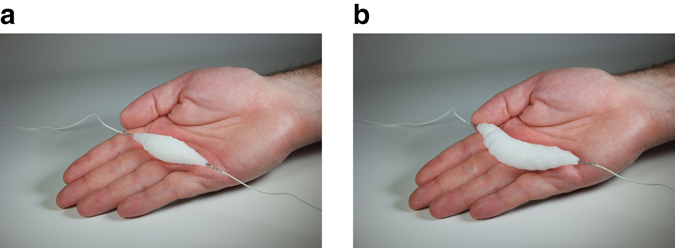
Soft artificial muscle. The muscle is composed of ethanol distributed throughout the solid silicone elastomer matrix. **a** Electrically actuated muscle including thin resistive wire in a rest position on a human hand. **b** Expanded muscle actuated (8 V, 1 A)

We show the material as an artificial muscle that can be electrically actuated using a thin resistive wire (Fig. 1a) and low power characteristics (8 V, 1 A) to exhibit significant expansion-contraction ability (Fig. 1b).

**Fig. 2:**
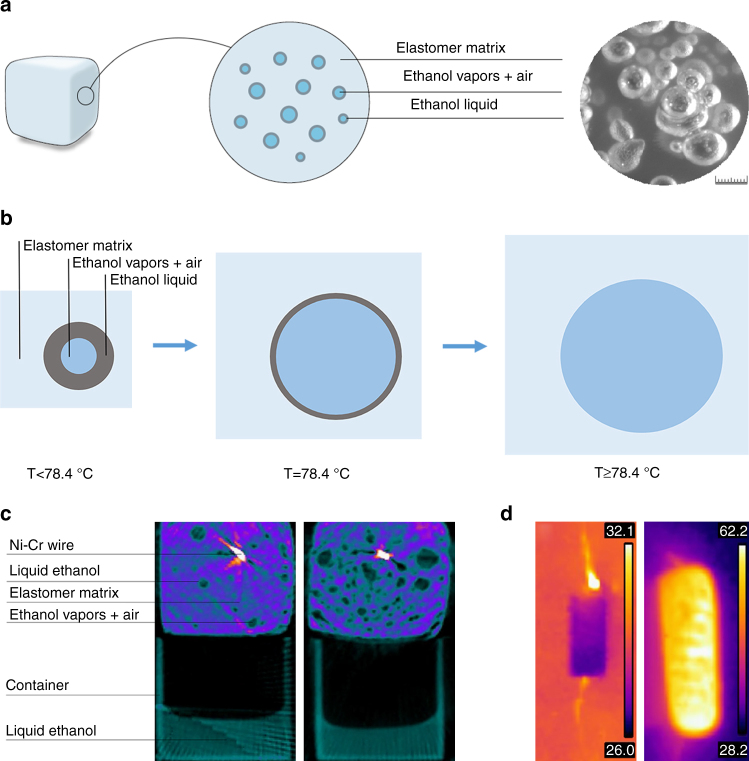
Structure and principle of operation of the soft composite material. **a** Microstructure: Illustration and a stereoscope image; *scale bar* is 1 mm. **b** Illustration of the expansion process on example of a single ethanol bubble. **c** Micro-CT images of the material cross-section at room temperature and during heating. **d** Infra-red images of the material as the heating starts and during expansion; the material is heated using Ni–Cr resistive wire

Due to mixing, ethanol is distributed throughout the silicone elastomer matrix in bubble-shaped pores and forms local pressure equilibrium with its vapors. During curing, ethanol vapors occupy air pockets distributed throughout the material, create new pores and lower the developing internal vapor pressure by expanding the pores until the equilibrium with the external environment pressure is achieved. Ethanol wets both the silicone elastomer gel and the cured solid (Supplementary Fig. 1). Thus, ethanol spreads on the inner walls of the bubbles and the remainder of the space inside them, if any, is occupied by ethanol vapors and air (Fig. 2a). Density of the material including 20 vol% ethanol was measured at 0.84 g cm^−3^. Upon heating the composite to a temperature of 78.4 °C, ethanol boils and the local pressure inside the bubbles grows, forcing the elastic silicone elastomer matrix to comply by expansion in order to reduce the pressure (Fig. 2b).

Micro-CT scans of the composite material cross-section before and after activation illustrate the expansion phenomena and provide insight into the material at room temperature and during the heating with a spiral-shaped resistive wire (Fig. 2c). To facilitate the interpretation of the micro-CT scans, the material specimens were placed on top of a plastic container with liquid ethanol.

Liquid ethanol evaporates with temperature, giving rise to internal pressure inside the bubbles, which results in slightly expanded silicone elastomer matrix. When ethanol passes the liquid–vapor phase transition, extreme volume change occurs and the silicone elastomer matrix significantly expands. With growth in local pressure, the boiling temperature increases and thus, continued heating to temperatures slightly higher than 78.4 °C is required for further expansion, until no liquid ethanol remains in the bubbles (Fig. 2b). Infrared radiation images of the material at room temperature and during expansion (using Ni–Cr spiral) are shown in Fig. 2d.

### Mechanical properties

**Fig. 3:**
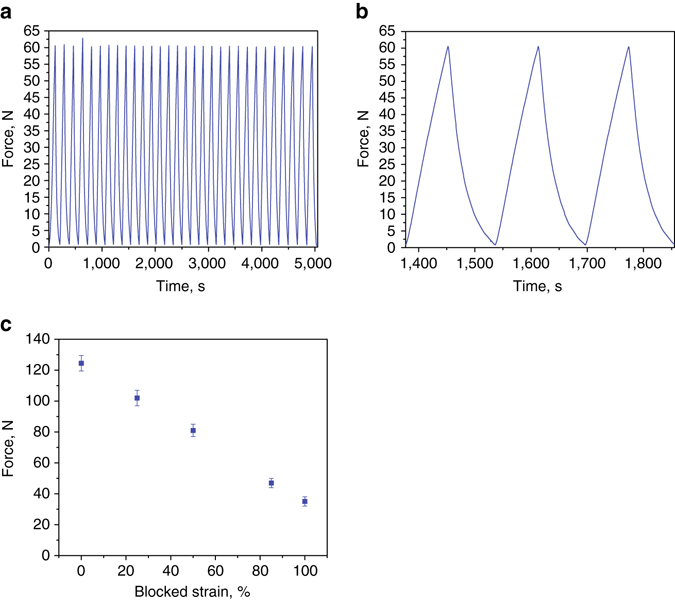
Force–strain characteristics of the material (15 V, 1 A). **a** 30 following cycles of loading to 60 N (blocked force; cylindrical specimen; diameter 15.1 mm, length 40 mm; weight 6 g). **b** Detailed view of three cycles in **a**. **c** Blocked force at various elongations for constrained cylindrical actuation; error bar relates to s.d. Specimen diameter 11.1 mm, length 25 mm; weight 2 g. Note that the actuation depends on heating and cooling rates

A maximal volume expansion of about 915% was measured at the temperature of 90 °C during controlled heating in a wide water bath (unconstrained volumetric expansion). We used an Instron machine to measure the blocked directional force and actuation stress characteristics of the material during its electrical actuation using resistive spiral-shaped wire at low power (15 V, 1 A). First, we aimed to demonstrate an ability of the composite to lift a weight 1000 times more than its own. The 6 g material showed an ability of repeated lifting of a weight of 6.1 kg, shown in Fig. 3a as 30 repeated cycles of loading to 60 N (blocked one-directional force). A detailed view of three loading–unloading cycles is shown in Fig. 3b. In a separate experiment, we measured maximal unidirectional force obtained at various blocked-strain levels in the 0–100% range. The maximal force for unstrained specimen weighing 2 g was about 120 N, which is equivalent to the actuation stress of 1.3 MPa and a maximal ability of lifting weight 6000 times larger than its own. Figure 3c shows that the force decreases with strain down to 35 N at 100% blocked strain. An extrapolation of the trend suggests that the material will reach strain limit of 140%, when allowed to expand in only one direction, as opposed to about 900% volumetric expansion when unconstrained. Both 3D-printed and cast specimens show similar actuation behavior (Supplementary Movie 4).

### Implementation in robotics

**Fig. 4:**
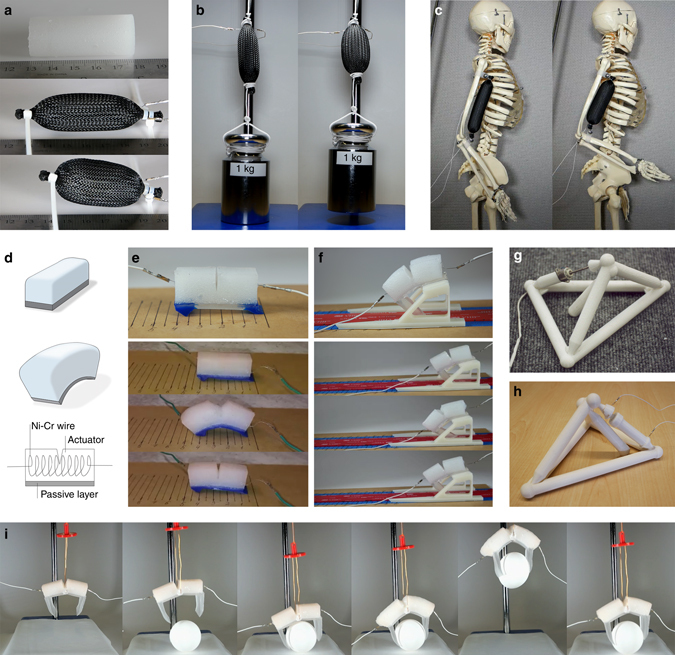
Implementation of the soft composite material as an actuator. **a** McKibben-type artificial muscle (soft composite material inside braided mesh sleeving) shows displacement of about 25%. **b** 13 g artificial muscle lifts the weight of 1 kg. **c** Soft artificial muscle implemented as a biceps lifting skeleton’s arm to 90° position at elbow (**a**–**c**: actuation powered at 45 W (30 V, 1.5 A)). **d** Design of the bi-morph bending actuator. **e** All-soft two-leg “worm” and its locomotion powered at 8 W (8 V, 1 A). **f** The sleigh robot and its locomotion powered at 8 W (8 V, 1 A). **g** Tetrahedral robot evolved and 3D-printed in 2000^34^ with embedded electrical motor. **h** The same robot with the soft composite material as an actuator embedded instead of the electrical motor. **i** Soft gripper lifting an egg (sequence from left to right; 8 V, 1 A)

We demonstrate the implementation of our composite material as an actuator in a variety of robotic applications (Supplementary Movie 2). First, we show a McKibben-type muscle. Our self-sufficient artificial muscle does not require any compressors or pressure-regulating equipment (Fig. 4a), and is capable of lifting weight much larger than its own (for example, a 13 g actuator lifts 1 kg in Fig. 4b). We demonstrate its use as a bicep, which contracts and pulls the lower arm up, causing it to bend at the elbow (Fig. 4c). The actuator is comprised of the composite material placed inside a braided mesh sleeving, fixed at the edges (Supplementary Fig. 3). The actuation is electrically driven using a spiral-shaped resistive wire (powered at 30 V, 1.5 A) passing inside the actuator. During the actuation, the composite material expands radially and contacts longitudinally, mimicking natural muscle behavior.

In addition, we designed an actuator consisting of our composite material attached to a layer of pure (unactuated) silicone elastomer, to create bimorph bending (Fig. 4d). The actuation is electrically driven using a resistive wire powered at 8 W (8 V, 1 A). We used this actuator in two models of robots: the all-soft two-leg “worm” (Fig. 4e), and a “sleigh” (Fig. 4f). During the actuation, the composite material expands and bends due to the constricting force at the vicinity of the passive silicone elastomer layer.

Figure 4i shows an electrically driven soft gripper, comprised of the composite material with two soft legs at the edges for grasping. Upon actuation, bimorph bending occurs and the legs move towards the item (e.g., a raw egg) and enable its grasping and lifting.

In addition to soft body locomotion and grasping, we demonstrate the ability of the artificial muscle to substitute electrical motor in an existing robot. We used a biomimetic robot produced using evolutionary algorithms^34^. The original robot^34^ (Fig. 4g) was 3D-printed and contained a small electrical stepper motor embedded in the upper bar. We reprinted the original robot and replaced the electrical motor with a detachable 3D-printed unit (Supplementary Fig. 4) including our composite material as the active material (Fig. 4h) embedded in a Teflon sleeve. We applied an electrical current (8 V, 1 A) through a resistive coil embedded inside the muscle, which exhibited directional expansion and acted as a piston moving the upper part of the bar forward. We demonstrate the locomotion of each robot in Supplementary Movie 2.

## Discussion

**Fig. 5:**
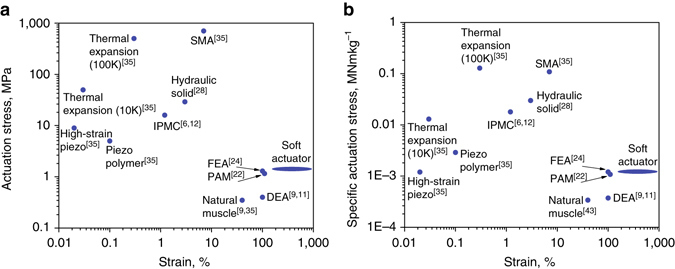
Comparative stress-strain charts for electrically driven actuators. **a** Actuation stress plotted against strain. **b** Specific actuation stress (actuation stress divided by density of the material) plotted against strain. Abbreviations: DEA- dielectric elastomer actuator, FEA- fluidic elastomer actuator, IPMC- ionic polymer-metal composite, PAM- pneumatic artificial muscle (McKibben actuator), SMA- shape memory alloy. The proposed material is labeled “Soft Actuator”. The ellipse designates a range of observed strains spanning from constrained unidirectional expansion of 140% to unconstrained volumetric expansion of 900%. For Thermal Expansion actuators, 10 and 100 K are the temperature change ranges in Kelvin degrees

The proposed soft composite material demonstrates a combination of high strain (up to > 900%) and correspondingly high stress (up to 1.3 MPa) at low density (0.84 g cm^−3^). Even at 100% strain the material develops stress of 0.4 MPa and is capable of lifting weight about 1700 times greater than its own. These characteristics place this material in previously inaccessible region of the actuator stress–strain charts (Fig. 5a). Our actuators are Pareto-undominated in specific actuation stress versus strain (Fig. 5b). We suggest that the strain limit of our material is the maximal strain of the silicone elastomer matrix (980%, according to the manufacturer). Along with its extremely low cost (laboratory cost of about 3 cent per gram), ease of fabrication, and environmental friendliness, these properties make this material an attractive solution where strain density is a critical factor.

**Fig. 6:**
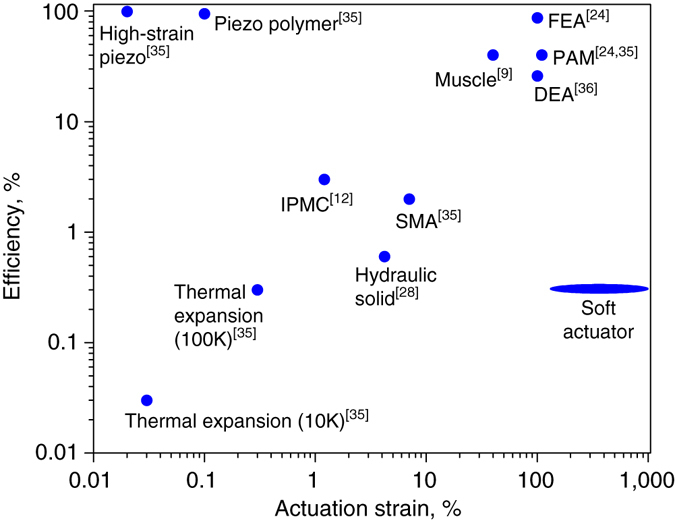
Maximal efficiency plotted versus actuation strain for various actuating methods. Our material is labeled as “Soft Actuator”. For Thermal Expansion actuators, 10 and 100 K are the temperature change ranges in Kelvin degrees. The elliptical shape denotes the range of strain possible ranging from purely linear expansion (140%) to full (unconstrained) volumetric expansion (900%)

Efficiency of the actuator heated by a resistive wire may be estimated as a ratio between the mechanical work produced per time, and the invested (consumed) electrical energy. Using data from Fig. 3, the consumed electrical power is a product of the applied DC voltage and current: 15 V·1A = 15 W. According to Fig. 3b, the time it takes the actuator to reach a force of 60 N was 70 s. The strain level at 60 N force may be estimated from Fig. 3c as 70%. Accordingly, linear expansion of the 40 mm long specimen was 0.7×40 mm = 28 mm = 0.028 m. Thus, the mechanical work done by the actuator may be calculated as a product of the force and the distance, namely 60 N·0.028 m = 1.68 J. The output power is 1.68 J/70 s = 0.024 W. Thus, the efficiency of the actuator is (0.024 W/15 W)·100% ≈ 0.2%. This value corresponds to heating caused by a single-coil of the resistive wire. In Supplementary Discussion and Supplementary Fig. 5 we show that using a wire of the same resistance in different designs (single-, double-, and triple-coiled wire), heating times may vary significantly. For instance, using triple-coiled wire shortens the heating time by 40%, allowing to increase the efficiency by this value. In this way, a simple change in a wire design, allowing more uniform distribution of the heat, may increase the efficiency of our material to about 0.3%. This value is comparable to the values for other thermal expansion actuators and for some shape memory alloys in the Ashby chart^35^. In Fig. 6 we show the efficiency plotted against the actuation strain for most of the existing actuation methods relevant for soft actuation, including materials (SMA, piezoelectric materials, etc) and devices (hydraulic and pneumatic setups).

It may be seen that actuation methods exhibiting high actuation strain (about 100%) along with high efficiency are based on either hydraulic or pneumatic (FEA, about 87%; PAM, about 40%) devices. For DEAs^36^, depending on the mode of operation, the maximal efficiency value of 26% was reported for the acrylic actuators (which are also superior to other DEAs in strain). However, such hydraulic and pneumatic solutions require external compressors and pressure-regulating equipment. In turn, DEAs require very high voltage for their operation. According to the chart, the next possible solution is SMAs; however, they show efficiency of about 2% and strains < 10%. This dichotomy highlights the state-of-the-art trade-off, in which a combination of reasonable efficiency and strain cannot be achieved using easily-operated material-actuators based on low voltage, but only by devices requiring external system for power conversion (a compressor or high-voltage converter). In contrast, the proposed material offers high strain directly from low-voltage source, typically available in untethered applications. We suggest that the high actuation strain, along with low cost and simplicity of preparation of the proposed soft material, merit further exploration of methods to improve its efficiency, which would then allow efficient material-actuator with very high strain.

Efficiency and operation of the proposed actuator material highly depends on heating and cooling rates. For resistive heating, higher current or more distributed heating networks are likely to provide faster material expansion. For cooling, an optimized design of the actuator geometry and the surface area may facilitate faster cooling rates. For example, a thin strip with large surface-to-volume ratio is likely to cool much faster than bulk material. In addition, active cooling solutions such as a Peltier Junction, or a liquid–flow cooling channels could also be used. In the latter, as silicone repels water (Supplementary Fig. 1), internal channels may be designed in the soft material for water-flow cooling. However, such solutions would require additional power and space on the potential robot/device.

**Fig. 7:**
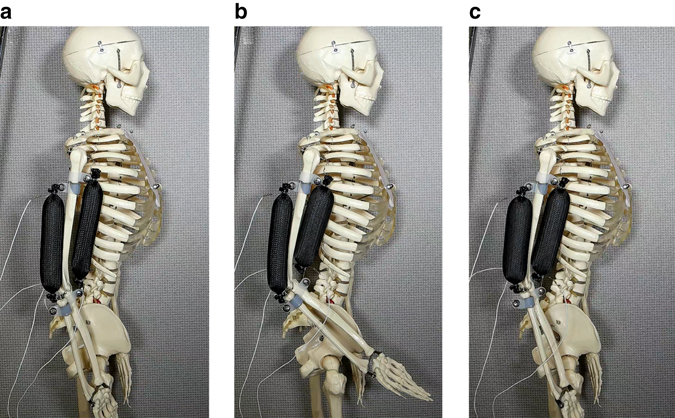
Agonist-antagonist soft actuator pair (20 V, 1 A). **a** Initial position of biceps and triceps actuators; **b** Actuation (bending the arm) by biceps; **c** De-actuation (bringing the arm to its initial position) by triceps. Actuators size: 20 mm diameter, 100 mm length. This setup reduced actuation time by a factor of 2.4 compared with a single actuator

Alternatively, we suggest a biology inspired solution using agonist-antagonist muscle pairs. It is well known that during the contraction of biceps to bend the arm at the elbow, triceps are relaxed, and vice versa. This feature may be used to significantly reduce the de-actuation time of the proposed actuator. Once one actuator (biceps) will complete its work to bend the arm, the task of bringing the arm back into its initial open position may be done by triggering the second actuator (triceps), instead of waiting for the cooling of the biceps (Fig. 7). In the Supplementary Movie 3 we show that this method may increase the actuation/de-actuation time 2.4 times.

To summarize, our work proposed a self-contained soft robust composite material, combining very high strain and reasonably high stress with low density, which is easily produced from bio-compatible components at a very low cost. This material-actuator may serve in a variety of applications, from traditional robotics to advanced bio-medical needs, and may enable a new kind of entirely soft robots.

## Methods

### Materials

We used platinum-catalyzed two-part silicone rubber Ecoflex 00-50 (Smooth-On, PA, USA) as a matrix material and ethanol ≥ 99.5% (Sigma Aldrich, MO, USA) as an active phase change material. Properties of the silicone rubber are shown in Table 1 below. Material preparation involves thorough hand-mixing of 20 vol% of ethanol with silicone elastomer (first with part A for about 2 min, then mixed with part B for about 2 min). The material is ready-to-cast and ready-to-print after the preparation. Room temperature curing of the cast or 3D-printed part takes up to 3 h. A commercially available 0.25 mm diamter Ni-chrome resistive wire was used for electrically driven heating of the artificial muscle (i.e., for the actuation). To comply with the expansion of the actuator material, a helical spiral shape was chosen for the Ni–Cr wire. The wire was hand-wound on an 8 mm screw driver shaft as shown in Supplementary Fig. 6.

**Table 1:** Properties of the silicone rubber Ecoflex 00-50 (manufacturer declared)

	Specific gravity (ASTM-D-1475)	Mixed viscosity (ASTM-D-2393)	Pot Life (ASTM-D-2471)	Cure time	Shore hardness (ASTM-D-2240)	Tensile strength (ASTM-D-412)	Elongation at break (ASTM-D-412)
Ecoflex 00-50	1.07 g cm^−3^	8000 cps	18 min	3 h	00-50	315 psi	980%

### 3D-Printing

Fabrication of the actuator using 3D-Printer was performed on a lab-made desktop 3D-printer capable of direct printing of two materials in one print. For optimal printing using 14 gauge syringe tip, the material was held in the mixing container for 15 min before printing. The maximum 3D printing resolution of 0.8  mm was achieved using 20 gauge syringe tip.

3D-printing of the robotic demonstrators was done on commercial FDM machines: Ultimaker 2 + , Ultimaker (Gendermalsen, Netherlands) for the sleigh robot (material: PLA), and uPrint, Stratasys (MN, USA) for tetragonal evolved robot (material: acrylonitrile butadiene styrene (ABS)).

### Characterization

Olympus SZ51 (Tokyo, Japan) stereoscope with analytic software was used for optical characterization of the actuator material. We used 30 × 10 × 3 mm size specimens and appropriate lighting conditions to obtain images with highest contrast. Micrometrix AccyPyc II1340 (GA, USA) pycnometer was used for density evaluation of the actuator material. Ramé-hart (NJ, USA) model 190CA goniometer was used for contact angle measurements. Headspace Gas Chromatography Mass Spectrometry (GC–MS) analysis was performed on the PerkinElmer Clarus SQ8C model (Waltham, MA, USA) apparatus. Micro-CT (Mediso, Boston, MA), a small-animal CT scanner with an image resolution of 100 microns, was used for x-ray computer tomography (CT) of the internal structure of the actuator material. Water bath experiment was conducted for evaluation of the maximal expansion of the actuator material. A desktop hot-plate was used to heat the water bath (borosilicate glass beaker) while the water temperature was continuously controlled by a K-type thermocouple connected to the Fluke 52-2 dual input digital thermometer ( ± 0.1 °C). The water level was monitored during the experiment and the volume was constantly evaluated. An Instron 8841 (MA, USA) machine was used for mechanical properties evaluation. For the blocked force experiment, the cylindrical muscle specimen (40 mm length, 15.1 mm diameter) was placed in a polytetrafluoroethylene (PTFE) cylinder, and inserted into a hollow aluminum cylinder with a closed top. The latter was attached to the upper static part of the Instron machine, while the specimen at the bottom faced a smaller aluminum cylinder connected directly to the load cell. The muscle was actuated by heating, provided using a Ni–Cr resistive wire and low power (15 V, 1 A). At heating, the muscle expanded and pressed against the bottom cylinder connected to the load cell, which detected and recorded the force. The experiment setup schematic is shown in Supplementary Fig. 7. Displacement against force was measured in a similar experiment, at which specimens with constant dimensions (25 mm length, 11.1 mm diameter) were placed in the PTFE tubes with various dimensions to enable expansion to a desired strain levels. Then blocked force was measured at the developed strain. Five specimens were tested in each testing set. The volume expansion and stress–strain experiments were conducted 24 h after the curing of the specimens.

### Data availability

All data are available from the authors upon reasonable request.

## Electronic supplementary material

Supplementary Information

Supplementary Movie 1

Supplementary Movie 2

Supplementary Movie 3

Supplementary Movie 4

Supplementary Software 1

## References

[1] Rus D, Tolley MT (2015). Design, fabrication and control of soft robots. Nature.

[2] Laschi C, Cianchetti M (2014). Soft robotics: new perspectives for robot bodyware and control. Front. Bioeng. Biotechnol..

[3] Kim S, Laschi C, Trimmer B (2013). Soft robotics: a bioinspired evolution in robotics. Trends Biotechnol..

[4] Trimmer B (2013). Soft robots. Curr. Biol..

[5] Trivedi D, Rahn CD, Kier WM, Walker ID (2008). Soft robotics: biological inspiration, state of the art, and future research. Appl. Bionics Biomech..

[6] Kim KJ, Shahinpoor M (2002). A novel method of manufacturing three-dimensional ionic polymer–metal composites (IPMCs) biomimetic sensors, actuators and artificial muscles. Polymer.

[7] Cianchetti M, Mattoli V, Mazzolai B, Laschi C, Dario P (2009). A new design methodology of electrostrictive actuators for bio-inspired robotics. Sensors Actuators B Chem..

[8] O’Halloran A, O’Malley F, McHugh P (2008). A review on dielectric elastomer actuators, technology, applications, and challenges. J. Appl. Phys..

[9] Mirfakhrai T, Madden JDW, Baughman RH (2007). Polymer artificial muscles. Mater. Today.

[10] Bar-Cohen, Y. EAP as artificial muscles – progress and challenges. in*Smart Structures and Materials* (ed. Bar-Cohen, Y.) 10–16 (International Society for Optics and Photonics, 2004). doi:10.1117/12.538698

[11] Galantini F, Carpi F, Gallone G (2013). Effects of plasticization of a soft silicone for dielectric elastomer actuation. Smart Mater. Struct..

[12] Shahinpoor M, Kim KJ (2001). Ionic polymer-metal composites: I. Fundamentals. Smart Mater. Struct..

[13] Huang WM (2010). Shape memory materials. Mater. Today.

[14] Ratna D, Karger-Kocsis J (2007). Recent advances in shape memory polymers and composites: a review. J. Mater. Sci..

[15] Laschi C (2012). Soft Robot Arm Inspired by the Octopus. Adv. Robot..

[16] De Greef A, Lambert P, Delchambre A (2009). Towards flexible medical instruments: Review of flexible fluidic actuators. Precis. Eng..

[17] Paik, J. Characterization of silicone rubber based soft pneumatic actuators.in*IEEE/RSJ International Conference on Intelligent Robots and Systems*4446–4453 (IEEE, 2013). doi:10.1109/IROS.2013.6696995

[18] Polygerinos P, Wang Z, Galloway KC, Wood RJ, Walsh CJ (2015). Soft robotic glove for combined assistance and at-home rehabilitation. Rob. Auton. Syst..

[19] Shepherd RF (2011). Multigait soft robot. Proc. Natl Acad. Sci.

[20] Tolley MT (2014). A resilient, untethered soft robot. Soft Robot.

[21] Marchese AD, Katzschmann RK, Rus D (2015). A recipe for soft fluidic elastomer robots. Soft Robot.

[22] Chou C-P, Hannaford B (1996). Measurement and modeling of McKibben pneumatic artificial muscles. IEEE Trans. Robot. Autom.

[23] Morin SA (2012). Camouflage and display for soft machines. Science.

[24] Sridar, S. et al. Hydro Muscle -a novel soft fluidic actuator. In *IEEE International Conference on Robotics and Automation (ICRA) *4014–4021 (IEEE, 2016).

[25] Bartlett NW (2015). A 3D-printed, functionally graded soft robot powered by combustion. Science.

[26] Ogden S, Klintberg L, Thornell G, Hjort K, Bodén R (2014). Review on miniaturized paraffin phase change actuators, valves, and pumps. Microfluid. Nanofluidics.

[27] Carlen ET, Mastrangelo CH (2002). Electrothermally activated paraffin microactuators. J. Microelectromech. Syst..

[28] Lipton JI, Angle S, Banai RE, Peretz E, Lipson H (2016). Electrically actuated hydraulic solids. Adv. Eng. Mater..

[29] Konishi S, Kawai F, Cusin P (2001). Thin flexible end-effector using pneumatic balloon actuator. Sens. Actuat. A Phys.

[30] Ma M, Guo L, Anderson DG, Langer R (2013). Bio-inspired polymer composite actuator and generator driven by water gradients. Science.

[31] Zhao Q (2014). An instant multi-responsive porous polymer actuator driven by solvent molecule sorption. Nat. Commun..

[32] Zhou Z, Li Q, Chen L, Liu C, Fan S (2016). A large-deformation phase transition electrothermal actuator based on carbon nanotube–elastomer composites. J. Mater. Chem. B.

[33] Altmüller R, Schwödiauer R, Kaltseis R, Bauer S, Graz IM (2011). Large area expansion of a soft dielectric membrane triggered by a liquid gaseous phase change. Appl. Phys. A.

[34] Pollack JB, Lipson H (2000). Automatic design and manufacture of robotic lifeforms. Nature.

[35] Huber JE, Fleck NA, Ashby MF (1997). The selection of mechanical actuators based on performance indices. Proc. R. Soc. A Math. Phys. Eng. Sci.

[36] Bigue J-PL, Plante J-S (2013). Experimental study of dielectric elastomer actuator energy conversion efficiency. IEEE/ASME Trans. Mechatron..

