# Supplementary material for: Soft material for soft actuators
Source: Nat Commun. 2017 Sep 19;8:596. doi: 10.1038/s41467-017-00685-3 (PMC5605691; doi:10.1038/s41467-017-00685-3)
Supplement: Supplementary file 1 — Supplementary Information [file 41467_2017_685_MOESM1_ESM.pdf]

Description of Supplementary Files

File Name: Supplementary Information
Description: Supplementary Figures, Supplementary Discussion and Supplementary References

File Name: Supplementary Movie 1
Description: How to make a soft robust composite actuator. This 9-minute video demonstrates in details how to prepare a soft compositematerial-actuator. A rectangular actuator is fabricated using basic ingredients, then tested 24 hours after the preparation.

File Name: Supplementary Movie 2
Description: Overview video of the soft composite material and its implementation. This 2.5-minute video demonstrates the soft composite-material-actuator as soft artificial muscle and its implementation in various modes of actuation.

File Name: Supplementary Movie 3
Description: Actuation/de-actuation using agonist-antagonist soft artificial muscle pair. This 40-seconds video demonstrates an alternative approach for increasing the de-actuation speed of the actuator by utilizing actuation of another actuator. We used artificial biceps-triceps agonist-antagonist muscle pair, prepared with our material, to demonstrate decrease of the de-actuation time. The video contains comparison of this approach with regular de-actuation by cooling.

File Name: Supplementary Movie 4
Description: 3D-printing and subsequent actuation of the soft material. This 2-minute video demonstrates 3D-printing and subsequent hot water bath (90°C) actuation testing of our soft material specimens. Two different actuation modes are shown: bi-morph bending of a specimen printed on a paper towel (paper serves as a passive layer), and volume expansion (constrained) of a specimen.

File Name: Supplementary Software 1
Description: The stl file of a mold suggested for preparation of the soft actuator.

Supplementary Discussion

Choosing the material system

The main goal of the work was to create a simple, cheap, fast and easily-prepared soft robust functional composite material for soft actuation, made of possibly bio-compatible (at least, user- and environment-friendly) components, allowing high strain at the 50÷100 ºC working temperature range.

This composite material should include elastic polymeric matrix and active fluid. In soft robotics, most widely used polymer type is silicone rubber. We searched for the room-temperature-curing matrix material with optimal mechanical properties (for achieving high strain along with bearing high stress), and having no odors or other possible application-limiting properties. Two such commercially available silicone rubber types were Sylgard of Dow Corning and Ecoflex series of Smooth-On. The first is actively used in bio-medical engineering, while the latter is marketed food-safe. We chose Sylgard 184 and Ecoflex 00-50 as the candidates for the matrix material.

Choosing the cheap and non-harmful active material for the given temperature range (the lower bar for preventing unwanted actuation, and the higher bar for safety and energy-saving) from a long list of potential candidates led us to water, ethanol- alcohol widely used in medicine, and the water/ethanol solution (used either for beverages or as medical solution). Chemical compatibility of the matrix and liquid materials was tested using contact angle measurement (wetting) experiment, followed by curing experiment.

The contact angle measurements (Supplementary Fig. 1) showed poor wetting of silicone rubbers by water, fair wetting by water/ethanol solution and excellent wetting by ethanol (with almost spreading for the Ecoflex 00-50 silicone rubber). This result suggests that silicone rubber will repel water, while ethanol will spread on the polymer matrix. For the curing experiment, we mixed the liquids with the silicone rubbers to test the mixing ability and curing. It is known that PDMS (lying in the base of these silicone rubbers) and ethanol are relatively compatible substances. Table 1 in the reference [1] shows that ethanol is PDMS-compatible for our application

with only slight swelling and low solubility. Swelling of the Ecoflex 00-50 elastomer components allowed us to mix the non-soluble ethanol and incorporate it inside prior to curing. The fact that ethanol perfectly wets the Eceflex 00-50 elastomer allowed to preserve ethanol after the curing, and ensure it spreads on the internal walls of the bubbles which formed during the mixing. Alternatively, Sylgard 184, which cures longer time at room temperature, showed no curing ability at the presence of ethanol. As a result, Ecoflex 00-50 was chosen as matrix material along with ethanol as an active fluid. We were able to mix various amounts of ethanol (0-33 vol.%) in the two-part platinum-catalyzed silicone elastomer Ecoflex 00-50 . Supplementary Fig. 2 shows Headspace Gas Chromatography Mass Spectrometry [2] (GC-MS) analysis results demonstrating ethanol content preserved in the material with different initial ethanol composition 15 days after preparation. Larger peak area (i.e., higher relative ethanol amount) was detected for higher initial ethanol content in the composite.

The materials with various ethanol content were compared for the expansion response to heat and it was found that specimens with 20 vol.% ethanol exhibit actuation response similar to that of the 33 vol.% ethanol, but with better repeatability and worklife. Thus, the material composition chosen for our composite was Ecoflex 00-50 matrix material with 20 vol.% ethanol. This material may be easily prepared by untrained personnel during 15 minutes and in 3 hours will fully cure. The material is comprised bio-compatible components. Laboratory cost of the material is 3 cents per gram.

It should be mentioned that the molecular size of ethanol and PDMS is comparable and may allow ethanol diffusion out of the elastomer matrix. Potentially we could use different liquids with larger molecular size to reduce or inhibit material depletion. However, inappropriate properties, such as low or high boiling point, hazardousness or harmfulness and incompatibility with silicone elastomer make several other liquids irrelevant for our actuator.

Key challenges and future research

The key challenges of our material are activation rate and shelf life. The responsiveness of our soft actuators depends on heat transfer rates. In the present work, we used Ni-Cr resistive wire because of its technical simplicity to demonstrate the material actuation. Alternatively, we were able to expand 6g of the composite material by 100% within 12s in a conventional microwave oven. The

heating times presented in this paper are based on a specific Ni-Cr resistive wire gauge (0.25 mm diam.) and thus, should not be taken as an absolute reference for material responsiveness. Faster heating, e.g. using higher current or more distributed heating networks, will result in quicker vaporization of the ethanol. For example, applying 1.5A instead of 1A resulted in significantly shorter actuation cycles (see examples in Supplementary Movie 2). Additionally, we used the Ni-Cr wire with two different total resistances, namely 10Ω and 20Ω, in three design configurations- a single spiral passing in the middle of the cylindrical composite specimen, two spirals connected in series placed at 180 degrees to each other at a diameter line, and three spirals connected in series placed at 120 degrees to each other. We monitored the heating rate of the composite material specimen to 80ºC (slightly above the boiling temperatue of ethanol, i.e. 78.4ºC) (Supplementary Fig. 5). Each measured time value was related to the longest time that took a composite specimen to reach 80ºC (namely, to the 10Ω single-spiral configuration) and presented as a relative time. The value were compared to the theoretical ones, which were calculated in the following way:

$$Q = m \cdot C \cdot \Delta T,$$

$$Q = \eta \cdot P \cdot t,$$

$$P = V \cdot I,$$

$$t = \frac{Q}{\eta \cdot P},$$

Where Q is the energy required to heat a material of with mass *m* and specific heat *C* (for Ecoflex matrix C=1558 $J \cdot kg^{-1} \cdot K^{-1}$ [3]), to a temperature difference of ΔT, η is the efficiency of the heater, *P* is the heating power, *V* is voltage, *I* is current, and *t* is the heating time. The calculation was done for heaters with 100% efficiency. As expected, twofold increase in the heating power resulted in twofold faster heating. The experimental results are in a good agreement with the calculated ones, showing reduction of about 50% in the heating time for the 20Ω as compared to the 10Ω wire of the same configuration. However, these theoretical calculations are based on the heating power and do not take into account the heater design. The experiment shows that better distribution of the heater throughout the composite material specimen results in significant reduction of the heating time at the same heating power. For example, for the 10Ω heater wire, the heating time decreased by 40% for the 3-spiral configuration, as compared to the single-spiral one.

Improving the heat conductivity of the silicone elastomer using additives with high thermal conductivity, such as graphene or metal powders, may shorten both heating and cooling times.

However, this approach should not only improve the heat conductivity of the composite, but allow retaining its functionality. Classical (percolation-related) solution suggests adding significant amount of additives, which affects negatively the expansion properties of the material. On the other hand, adding amounts that allow actuation does not change the heating-cooling rates. For example, adding 40 vol.% of copper makes the material completely non-expandable, while adding 1 vol.% copper does not affect the expansion abilities, but also does not change the heating-cooling rates. Heating rate of such material fits the behavior described in the Supplementary Fig. 5. Alternative solutions include utilizing the form-factor of the actuator and surface-to-volume ratio. Optimized actuator geometry and surface area may facilitate faster cooling rates. Active cooling solutions such a Peltier Junction could also be used although they would require additional power and space. Non-uniform heating using arbitrarily configured resistive networks can be used to produce arbitrary actuation kinematics by design.

A second challenge to our material is the evaporation of ethanol from the actuator. A material specimen left in open air will begin to degrade within hours of manufacturing and will eventually "dry up" and lose the ability to actuate in less than a week. To maintain longer shelf life and consistent performance, our material needs to be encapsulated in an ethanol-tight "skin". The actuator shown in Supplementary Fig. 7 was embedded in a Teflon tube, though other skins and encapsulations are possible. Storage in airtight containers, such as molds sealed with lids, may extend the shelf life to several months.

As in all other soft actuator systems, control remains challenging because of the inherent difficulty of modeling and predicting large deformations in soft solids. Until modeling has reached adequate fidelity, control must be done using sensors and feedback.

Shelf life and storage conditions

Shelf life of the material may be significantly extended by its storage in airtight containers, such as molds sealed with lids. This way, negligible degradation of <0.01g per month may occur and the specimens will preserve their actuation ability. We tested the materials kept in this condition, and they showed the anticipated actuation performance. We were able to keep specimens for at least 4 months. We did not yet perform storage experiments longer than 4 months."

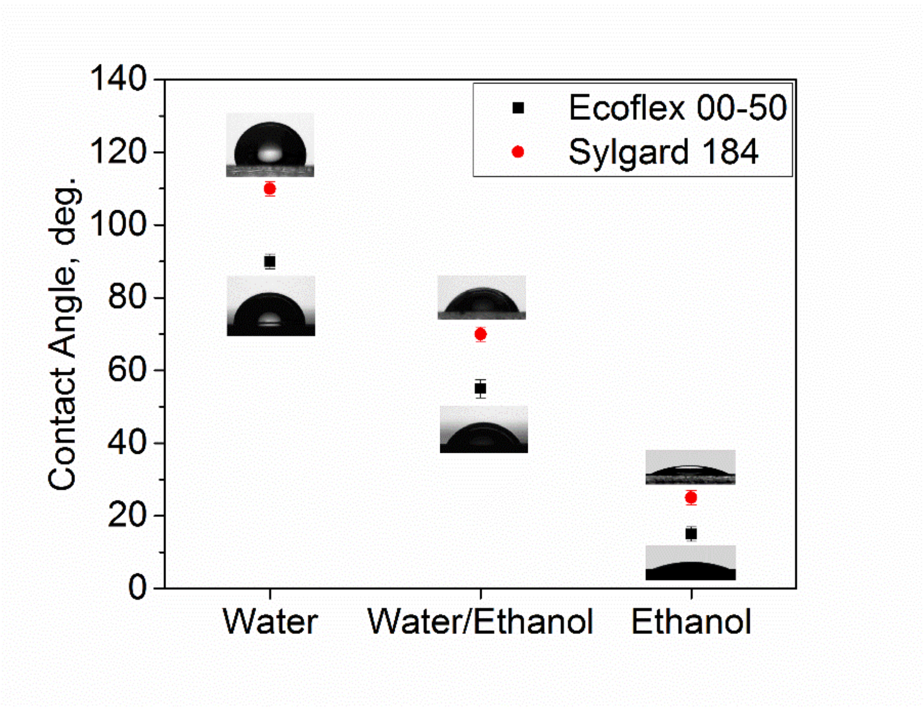

Supplementary Fig. 1. Wetting angle of chosen liquids (water, water/ethanol 60/40 vol.%, ethanol) on chosen polymer matrix types. Water, ethanol and water/ethanol solution were chosen as candidates for serving as active fluid at the 50÷100ºC temperature range. Most widely used in soft robotics polymers, namely two silicone rubber types, were tested for wetting by the chosen liquids (and for curing ability at the presence of these liquids). The plot shows non-wetting of both silicone rubber types by water and very good wetting by ethanol with almost spreading on the Ecoflex 00-50 substrate. Along with the ability to cure at the presence of ethanol (separate experiment- not demonstrated here), perfect wetting of the food-grade Ecoflex 00-50 silicone rubber by ethanol allowing to mix ethanol with the silicone rubber gel and preserve it inside during the curing spread on the internal walls of the bubbles, creating a user- and environment- friendly soft functional composite material.

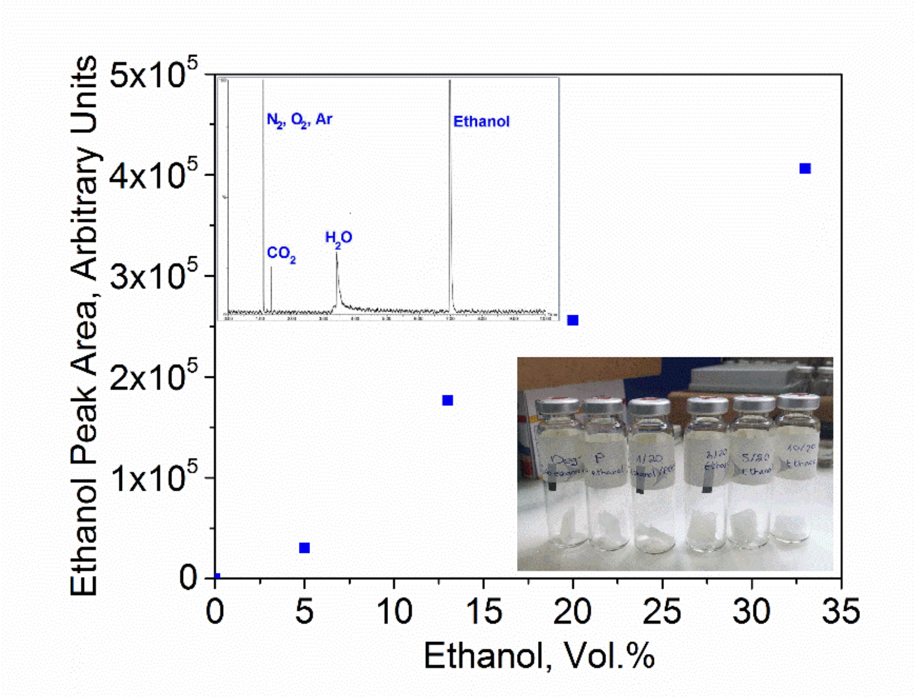

Supplementary Fig. 2. Ethanol peak area (as measured by GS-MS) as a function of ethanol content in the composite material specimen. Typical GC-MS chromatogram zoom-in shown in top left corner refers to the sample with initial ethanol concentration of 13vol.%, 15 days after preparation, after heating to 80°C for 30 min., X axis is retention time in min., Y axis is intensity in %. Vails with composite material specimens before the Headspace Gas Chromatography Mass Spectrometry (GC-MS) analysis experiment are demonstrated in bottom left corner.

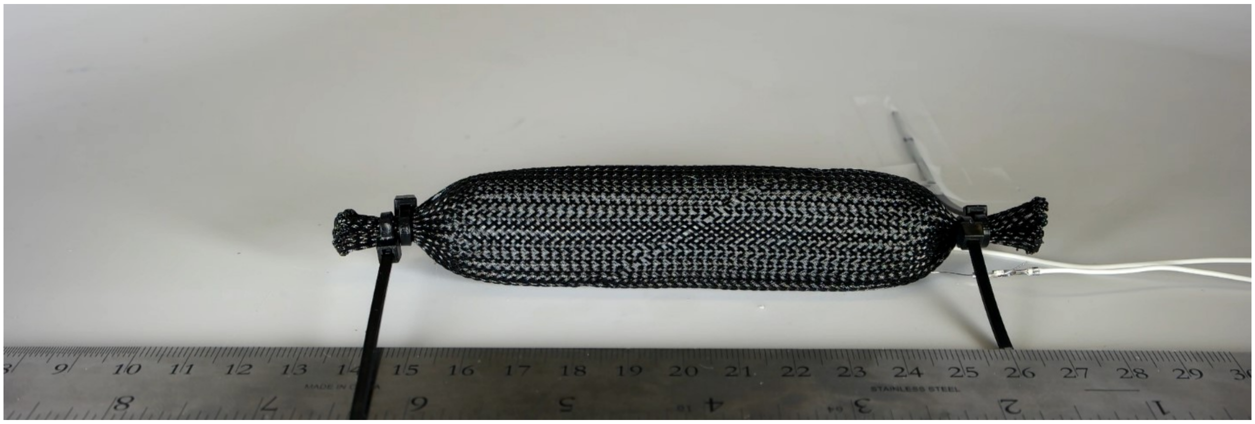

Supplementary Fig. 3. Soft robust composite material as a McKibben-type artificial muscle. The actuator is easily prepared by placing the soft composite material in a braided mesh sleeving and closing it using a zip tie and does not require any additional equipment. The actuation is performed using a spiral-shaped resistive wire with various possible spiral configurations.

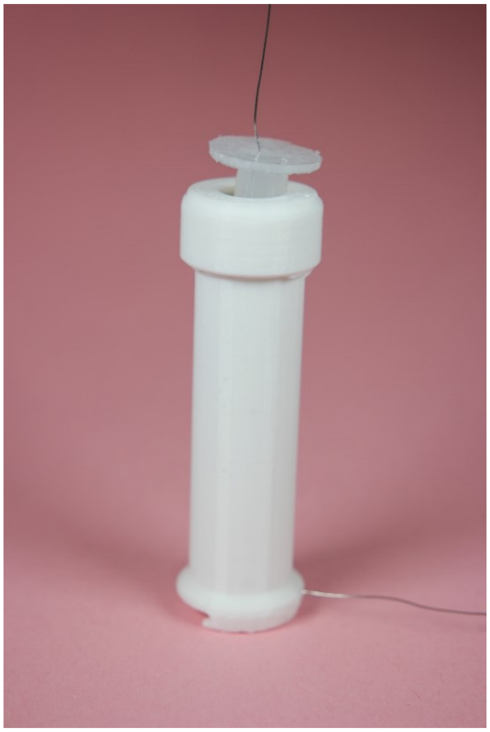

Supplementary Fig. 4. Retractable actuator unit with the soft composite material substituting the electrical motor in the evolved robot. The unit consists of the 3D-printed tube with open top side, the cast soft composite material, cast silicone-rubber-made piston, spiral-shaped Ni-Cr resistive wire passing inside the cast composite material. The piston simply attaches by clicking to the upper part of the robot body using a small 3D-printed adapter (not shown on the image), while the tube bottom has a special shape for attachment (clicking) to the bottom part of the robot. The unit may be easily attached to the robot or exchanged just in 2-3 seconds. When actuated using low power, the Ni-Cr wire heats up the composite material, which pushes up the piston, which in turn pushes up the adjacent robot part. Reverse motion is enabled by contraction of the soft composite material, retracting the piston and the adjacent robot part.

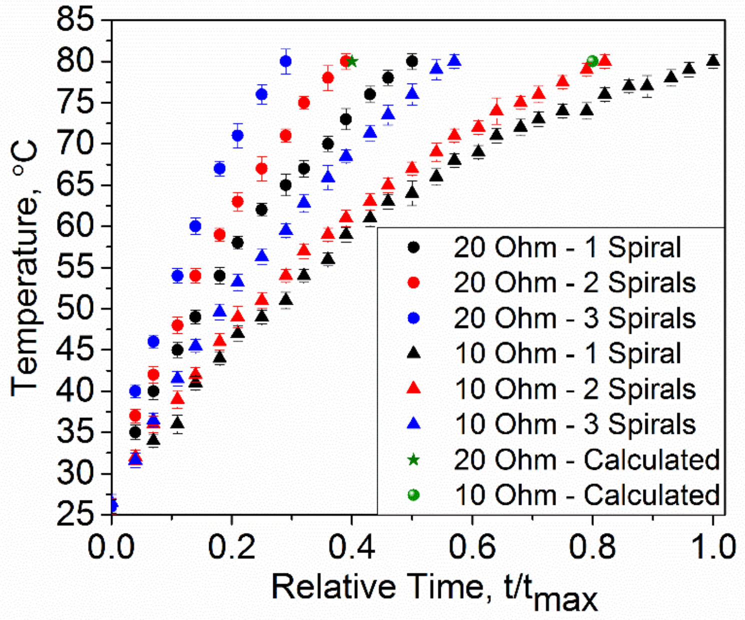

Supplementary Fig. 5. Effect of configuration of the resistive wire on heating of the composite material. Two total wire resistance values of 10 Ω (10V, 1A) and 20 Ω (20V, 1A) (i.e., two total wire lengths) with three wire configurations for each were used: 1 spiral (in the middle of the composite material); 2 spirals (at 180 deg to each other; connected in series); 3 spirals (at 120 deg; connected in series). Specimen dimensions are 20 mm in diameter and 50 mm in height. Relative time is the measured time value for each configuration divided by the longest measured time for reaching 80ºC (which was 140 s for the 1-spiral configuration). Theoretical values were calculated for heater with 100% efficiency. The trend shows that for the heater same resistance, the heating time may be significantly reduced using different heater design.

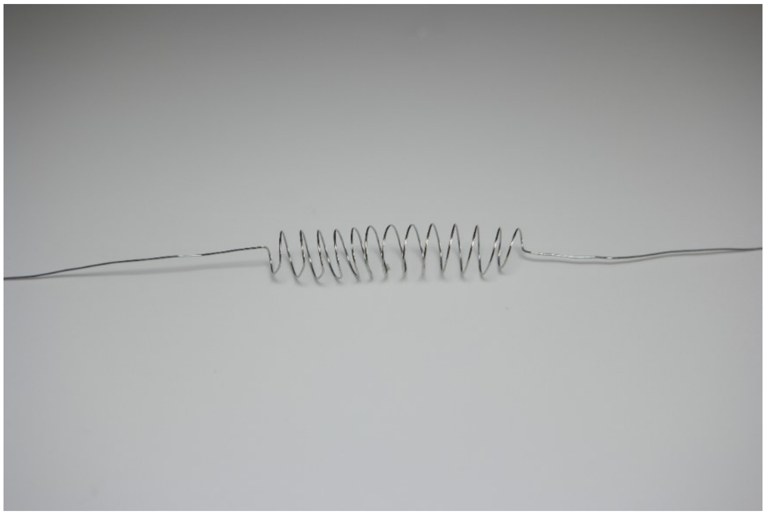

Supplementary Fig. 6. Spiral-shaped Ni-Cr resistive wire (1-spiral configuration). The shown coil was made by hand by winding 0.25mm-diamater wire over an 8mm-diamater screw driver shaft. This coil with 12 windings was then inserted into the mold while casting the actuator.

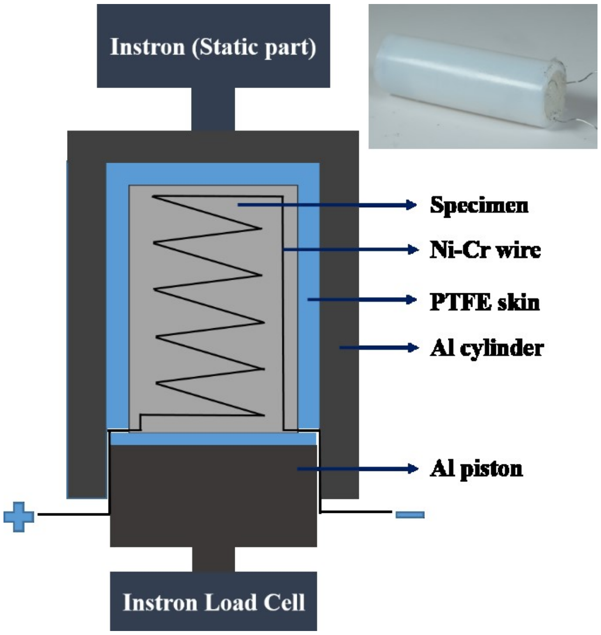

Supplementary Fig. 7. Schematic setup of the composite material testing in the Instron apparatus (blocked force tests) and a specimen image. The setup includes an aluminum tube with one open side, an aluminum piston, PTFE skin in the shape of a tube with open top and a PTFE lid. Ni-Cr resistive wire passes inside the cylindrical specimen for easy actuation. The bottom part of the aluminum tube is attached to the static part of the Instron machine, while the open side of the tube faces the aluminum piston, attached to the Instron load cell. When actuated, the resistive wire heats up the soft composite material, which applies force on the tube and the piston, allowing measurement by the load cell. To avoid shorts between the wire and the metal parts, the aluminum tube and piston are isolated using electrical insulator tape.

Supplementary References

[1] Lee, J. N., Park, C. & Whitesides, G. M. Solvent compatibility of poly(dimethylsiloxane)-based microfluidic devices. *Anal. Chem.* **75,** 6544–54 (2003).

[2] Snow, N. H. & Slack, G. C. Head-space analysis in modern gas chromatography. *TrAC Trends Anal. Chem.* 21, 608–617 (2002).

[3] Cabibihan, J.-J., Joshi, D., Srinivasa, Y., Chan, M. A. & Muruganantham, A. Illusory Sense of Human Touch from a Warm and Soft Artificial Hand. *IEEE Trans. Neural Syst. Rehabil. Eng.* **23,** 517–527 (2015).
